# A New Operative Procedure Facilitating the Adaptation of Artificial Dentures*Read before the Section on Stomatology at the Sixty-Seventh Annual Session of the American Medical Association, Detroit, June, 1916. Reprinted from the* Journal of A*. *M. A.*, January 20, 1917.

**Published:** 1917-02

**Authors:** H. A. Potts


### A NEW OPERATIVE PROCEDURE FACILITATING THE ADAPTATION OF ARTIFICIAL DENTURES.*

BY H. A. POTTS, M.D.

Authorities on prosthetic dentistry agree that, before taking an impression, a careful and comprehensive study of the mouth from both the physiologic and the pathologic aspects should be made. In an edentulous mouth, the thickness and firmness of the soft tissues are noted, as well as stomatitis, leukoplakia and other diseased conditions, if they are present. The bony ridges are studied with regard to undue pressure which may be brought to bear on them.

If natural teeth are to be retained, they and their supporting structures must be restored to health, and in either case the attachments of muscles are noted. All of these conditions are studied in order that the denture may meet the following requirements, namely, that they may be useful, look well and be comfortable (Prothero). Primarily all of these depend on their retention which is made possible by two physical factors, adhesion, which plays the lesser rôle, and atmospheric pressure.

On account of the varying density and compressibility of the different tissues in the same mouth, both impression

---

* Read before the Section on Stomatology at the Sixty-Seventh Annual Session of the American Medical Association, Detroit, June, 1916. Reprinted from the *Journal of A. M. A.*, January 20, 1917.

and model are scraped in order that a secure adaptation of the plate to the tissues may be maintained under all the various movements and different degrees of stress to which the plate may be subjected.

While the foregoing applies to the normal mouth, the same adaptation, adhesion and atmospheric pressure apply to the abnormal mouth. To correct the abnormal mouth, surgical procedures are resorted to, namely, the removal of loose fibrous or spongy tissues, the cutting of fibrous bands, the excision of spicules of process, or well defined osteoid growths which occur in the palate, labial, lingual or buccal regions of the jaws.

The removal of exposed, projecting process after extraction should be more frequently done than is our habit.

There is another quite common condition which can be remedied by surgical interference and which, so far as I can judge from a review of the literature and conversation with some of the leaders in dental thought, has not been pointed out, and that is the practical elimination of bony undercuts in the upper jaw. If this is done, the making of metal dies and the swaging of metal plates would be simplified, and the making of cores unnecessary.

A conformation which requires the slipping in of a plate as a shoe does not favor retention, but rather militates against it, as the chief factor in retention is atmospheric pressure.

When such a plate, the labial and buccal borders of which are forced into undercuts at one or more places, is subjected to the forces of mastication, the upper borders are carried away from the soft tissues, and air passes beneath the plate with its consequent release.

The study of the case here reported and the excellent result obtained by surgical means on the bony process seems to justify its more frequent performance.

The patient, a woman, aged fifty, had long since lost all upper teeth posterior to the cuspids, and mastication on the remaining six anterior ones, together with some destruction of peridental membrane, had resulted in a marked protusion. Her dentist, Dr. F. H. Stafford of Chicago, extracted the remaining teeth and proposed to make an upper denture, but found it quite impossible.

He referred her to me for surgical intervention. There is a very marked protrusion of the alveolar process which was covered with quite normal gum tissue and mucous membrane.

Two incisions along the alveolar ridge, extending from the second bicuspid region on the one side to the corresponding point on the other were made, which, being "quarter moon" shaped, eliminated the gum tissue which was between the teeth and left two cleanly cut borders of the flaps. A medium vertical incision extending from the reflection of the mucous membrane joined the other incision at right angles. The resulting flaps, together with the periosteum, were reflected.

By means of rongeur forceps and large bud shaped burrs the outer plate of bone was removed from the first bicuspid socket on the one side to the corresponding one on the other side. The thickness of bone removed varied, in the bicuspid region being the outer plate only, but the remainder extended to the posterior walls of the alveoli.

The incision along the alveolar border as well as the vertical one was closed with interrupted horsehair sutures; there was primary union within ten days. Impressions of the new normal mouth were taken, and a perfectly satisfactory denture constructed.

